# A leader cell triggers end of lag phase in populations of *Pseudomonas fluorescens*

**DOI:** 10.1093/femsml/uqac022

**Published:** 2022-11-02

**Authors:** Maxime Ardré, Guilhem Doulcier, Naama Brenner, Paul B Rainey

**Affiliations:** Laboratoire Biophysique et Évolution , CBI, ESPCI Paris, Université PSL , CNRS, 75005 Paris, France; Laboratoire Biophysique et Évolution , CBI, ESPCI Paris, Université PSL , CNRS, 75005 Paris, France; Network Biology Research Laboratories, and Department of Chemical Engineering, Technion–Israel Institute of Technology, Haifa, Israel; Laboratoire Biophysique et Évolution , CBI, ESPCI Paris, Université PSL , CNRS, 75005 Paris, France; Department of Microbial Population Biology, Max Planck Institute for Evolutionary Biology, Plön, Germany

**Keywords:** high-throughput millifluidics, growth dynamics, collective behavior, extreme value theory, microbial population biology

## Abstract

The relationship between the number of cells colonizing a new environment and time for resumption of growth is a subject of long-standing interest. In microbiology this is known as the “inoculum effect.” Its mechanistic basis is unclear with possible explanations ranging from the independent actions of individual cells, to collective actions of populations of cells. Here, we use a millifluidic droplet device in which the growth dynamics of hundreds of populations founded by controlled numbers of *Pseudomonas fluorescens* cells, ranging from a single cell, to one thousand cells, were followed in real time. Our data show that lag phase decreases with inoculum size. The decrease of average lag time and its variance across droplets, as well as lag time distribution shapes, follow predictions of extreme value theory, where the inoculum lag time is determined by the minimum value sampled from the single-cell distribution. Our experimental results show that exit from lag phase depends on strong interactions among cells, consistent with a “leader cell” triggering end of lag phase for the entire population.

## Introduction

When bacteria encounter new environmental conditions, growth typically follows four phases: a lag phase, during which bacteria acclimate, but do not divide; an exponential phase, during which cells multiply; a stationary phase, where nutrient exhaustion causes cessation of growth; and finally a death phase, during which cells may lyse. In a fluctuating environment, each phase can play an important role in population persistence. The lag phase has particular significance because of both benefits (enhanced growth) and eventual costs (sensitivity to external stressors) associated with the resumption of growth (Moreno-Gámez et al. 2020, Şimşek and Kim 2019). Moreover, the time to resumption of growth—and controlling factors—has implications for the entire field of microbiology (Monod 1949), but especially for infection caused by pathogens and for food safety (Bertrand and Margolin 2019, Swinnen et al. 2004, Pérez-Rodríguez 2014).

Despite its discovery more than 100 years ago (Müller 1895), cellular and molecular details defining the lag phase, factors triggering resumption of growth, and contributions to fitness, are not well-understood. This is largely a consequence of the difficulties associated with experimental quantification of the dynamics of populations founded by small numbers of cells. Nonetheless, advances over the last decade have shown that bacteria in lag phase are transcriptionally and metabolically active (Rolfe et al. 2012), that lag phase is a dynamic state, that single cells are heterogeneous in time to resume division (Julou et al. 2020, Moreno-Gámez et al. 2020, Şimşek and Kim 2019), and that numerous factors affect lag phase duration (Nikel et al. 2015, Bertrand and Margolin 2019, Basan et al. 2020).

Arguably the most intriguing aspect of lag phase biology is the apparent inverse relationship between the number of cells in the founding population and duration of lag phase—often referred to as the “inoculum effect.” First reported in 1906 (Rahn 1906), the relationship has been shown to hold for a number of different bacteria (Penfold 1914, Lodge and Hinshelwood 1943, Lankford et al. 1966, Kaprelyants and Kell 1996), although there exist few recent quantitative investigations. In certain instances, the inoculum effect is observed only under specific culture conditions (Kaprelyants and Kell 1996, Augustin et al. 2000).

Factors controlling the inoculum effect are of special interest (Dagley et al. 1950, Lankford et al. 1966, Halmann and Mager 1967, Augustin et al. 2000, Swinnen et al. 2004, Bertrand and Margolin 2019). Given that bacterial cells are typically variable in many of their properties, the simplest explanation (Explanation I) posits that population lag time is determined by the set of cells with the shortest time to first division. Accordingly, the larger the founding population, the more likely it is that the inoculum contains cells on the verge of division, with these cells contributing disproportionately to the resumption of population growth.

An alternate explanation is that resumption of growth depends on interaction among founding cells (Explanations II and III), e.g. via production of an endogenous growth factor: once a critical threshold concentration is achieved growth resumes, and the larger the inoculum the sooner this happens. Evidence in support of such control derives from analysis of *Bacillus* (Lankford et al. 1966), *Francisella* (formerly *Pasturella*) *tularensis* (Halmann and Mager 1967), *Micrococcus luteus* (Votyakova et al. 1994), and *Aerobacter aerogenes* (Dagley et al. 1950).

In instances where exit from lag-phase is determined by interactions among founding cells, models have assumed that all cells are equal contributors to the production of growth activating factors (Explanation II; Lankford et al. 1966, Kaprelyants and Kell 1996). However, an alternative possibility exists, namely, that population lag time is set by the activity of a single “leader cell” that triggers resumption of growth for the entire population of cells (Explanation III). Distinguishing among competing hypotheses requires precision measurements of population growth, high levels of replication, ability to control inoculum size, and crucially, knowledge of the distribution of lag times for single cells.

Here, we use a millifluidic droplet device in which the growth dynamics of hundreds of populations founded by different numbers of *Pseudomonas fluorescens* cells were followed in real time. Our data confirm that lag phase shortens with inoculum size increase and provide a quantitative characterization of the effect on: average, variance and shape distribution of lag times values for various controlled inoculum size. We demonstrate that these statistical properties follow extreme value theory (EVT), where population lag time is determined by the minimum value sampled from the single cell distribution. Additionally, we show that the inoculum effect cannot be explained by a sweep initiated from a small number of cells, but rather involves the parallel growth of many lineages. These results suggest that exit from lag phase depends on strong interactions among cells, consistent with a leader cell triggering end of lag phase for the entire population. And we derive the scaling laws that allows prediction of bacterial population lag time as a function of inoculum size.

## Methods

### The strain

The ancestral strain of *P. fluorescens* SBW25 was isolated from the leaf of a sugar beet plant at the University of Oxford farm (Wytham, Oxford, UK; Silby et al. 2009). The strain was modified to incorporate, via chromosomally integrated Tn*7*, the gene GFP-mut3B controlled by an inducible Ptac promoter.

### Preparation of cells

*Pseudomonas fluorescens* SWB25 was grown in casamino acid medium (CAA). CAA for 1 l: 5g of Bacto Casamino Acids Technical (BD ref 223120), 0.25 g MgSO_4_·7H_2_O (Sigma CAS 10034-99-8), and 0.9 g K_2_HPO_4_ (Sigma CAS 7758-11-4). Prior to generation of droplets, SBW25 was grown from a glycerol stock for 19 h in 5 ml of CAA incubated at 28°C and shaken at 180 rpm. At 19 h, this stationary phase culture was centrifuged at 3743 RCF for 4 min and the supernatant removed from the pellet. The pellet was then resupended in 5 ml of sterile CAA. It was then centrifuged and resuspended one further time in order to prevent any interference of a growth-activator that may come from the supernatant of the overnight culture. The washed culture was then adjusted to OD 0.8 with CAA and mixed 1:1 in volume with autoclaved 30% v/v glycerol. A volume of 100 μl aliquots were pipetted in 1 ml eppendorf and frozen at −80^°^C. After freezing, one aliquot was taken to measure viable cells by plating on agar. We found 1.62 × 10^8^ cell ml^−1^.

### Generation of droplets with a range of inoculum sizes

Each experiment, with a range of inoculum sizes was prepared as follows. One frozen aliquot was thawed and diluted in 6 Falcon^©^ tubes with a final volume of 4 ml of sterile CAA. The frozen aliquot was diluted, with appropriate intermediate dilutions, in the tubes respectively by 7.04 × 10^4^, 1.76 × 10^4^, 4.4 × 10^3^, 1.1 × 10^3^, 2.75 × 10^2^, and 6.875 × 10^1^ to obtain, respectively, 1, 4, 16, 64, 256, and 1024 cells per droplet (on average). We completed the dilutions from frozen stock by adding 29.1 μl, 29 μl, 28.6 μl, 27.3 μl, 21.8 μl, and 0 μl, respectively, of sterile 60% v/v glycerol. This step is very important to balance the glycerol coming from the frozen stock and ensure that all the tubes have precisely the same composition of medium (Figure S2, Supporting Information). We then added 50 μl of sterile IPTG (100 mM) to each sample. Each dilution was then pipetted in wells (250 μl per well) of a 96-well microtitre plate before proceeding to generate droplets using the Millidrop Azur device. Droplets have a volume of 0.4 μl, which yields, with our dilutions, a range of inoculum sizes as follows: 1, 4, 16, 64, 256, and 1024 cells per droplet. We generated 40 replicate droplets for each population of a given founding inoculum size, except for populations founded by 1024 cells, which for technical reasons were restricted to 30 replicates.

### The inoculum of droplets follows a Poisson distribution

Importantly, the inoculum size in each droplet is controlled by the Poisson process intrinsic to the formation of droplets from the 96-well plates. The inoculum size that we report is, thus the average of the corresponding Poisson distribution. In particular, the variance of the number of cells between the droplets is equal to the average inoculum.

### Generation of droplets founded by a single cell

To generate droplets with an inoculum of a single cell per droplet, we diluted in sterile CAA a frozen aliquot by 7.04 × 10^4^, added 29.1 μl of sterile 60% v/v glycerol, and 50 μl of sterile IPTG (100 mM). We generated 230 droplets in the Millidrop Azur device, which yielded 156 droplets that grew due to the Poisson process inherent to the sampling process.

### Log-normality of single-cell lag times

The cumulative distribution function (CDF) for single-cell lag times is displayed in Fig. 2(B), and is described by a lognormal fit. To quantify the goodness of fit, we used the Shapiro–Wilk test with the null hypothesis that a sample log θ_1_,..., log θ_*n*_ is derived from a normal distribution. The null hypothesis was tested with significance level alpha 5% and gave a *P*-value of 0.840 indicating that we can not reject the lognormality of the single-cell lag time distribution. In addition, a quantile-to-quantile plot of single-cell lag time against a lognormal distribution is shown in Figure S11 (Supporting Information).

**Figure 1.**
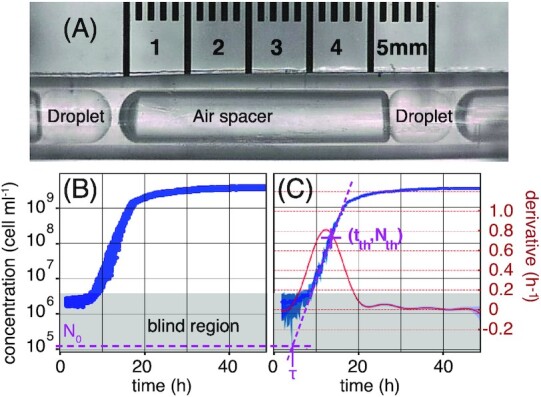
Bacterial population growth in droplets. Subfigure (A) shows two droplets of 0.4 μl are separated by an air spacer (to prevent droplet coalescence) inside the tube of a millifluidic machine. Droplets are prepared by “sipping” samples from a 96-well plate. Typically, 230 droplets are produced from six seed cultures that differ solely in the number of founding cells (the *inoculum*). Each seed culture delivers 40 replicate droplets, but for technical reasons that last delivers 30 replicates. Droplets move back-and-forth, via changes in pressure, passing in front of a fluorescence detector every ∼18 min. *Pseudomonas fluorescens* SBW25 cells express GFP from a chromosomally integrated reporter, allowing changes in biomass to be determined based on intensity of the fluorescent signal (excitation at 497 nm emission at 527 nm). Signal intensity is calibrated to cell density by plate counting (Figure S8, Supporting Information). The range of detection extends from 4 × 10^6^ to 5 × 10^9^ cells ml^−1^ (1.6 × 10^3^ to 2 × 10^6^ cells per droplet). The gray area in subfigures (B) and (C) denotes the region where bacterial density is below the threshold of detection. (B) Fluorescent signal across time from 40 replicate populations (in semi-logscale) in droplets prepared from the same seed culture. The average inoculum in each droplet is *N*_0_ = 1.6 × 10^5^ cell ml^−1^, or 64 cells per droplet (this concentration is marked by the purple dashed line that goes across (B) and (C)). In this example, the signal exceeds the detection threshold at ∼7 h, by which populations are in exponential growth phase. At ∼20 h, stationary phase is reached, marked by cessation of growth. (C) A single time series showing population growth within a single droplet coming from the set of replicates shown in (B). The left *y*-axis is shared between these two plots. The blue line depicts cell density derived using DropSignal (Doulcier 2019) and the shaded area represents the standard deviation (SD). Population lag time is inferred as described in text. The purple dotted line crossing *N*_*th*_ = 1.6 × 10^8^ cells ml^−1^ (64 000 cells per droplet) extrapolates the exponential growth back to its intersection with the inoculum density (purple horizontal dotted line), giving τ ≈ 5 h. The red line gives the derivative of the time series, with shaded SD, and corresponding right *y*-axis in red.

**Figure 2.**
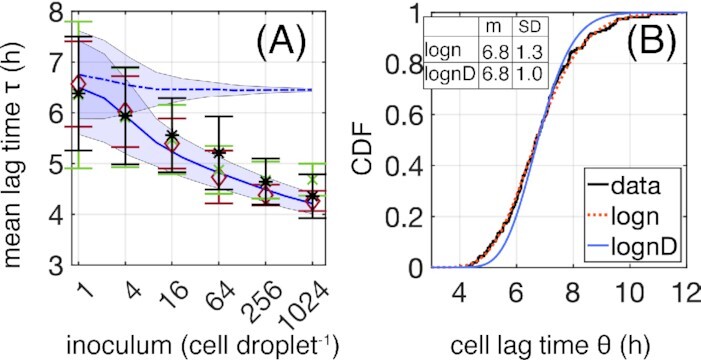
Quantitative data on lag-times are consistent with a strongly cooperative exit from lag phase. (A) Population lag time τ as a function of inoculum size for three independent experiments (colors). Symbols are the mean lag times over droplets with a given inoculum size, with error bars denoting the standard deviation (SD). The data are compared to two models (blue lines—average, shaded blue—SD). (B) Cumulative distribution (CDF) of single-cell lag times (θ) from 156 droplets inoculated with a single bacterium on average (dark line). The *y*-value gives the probability that cell lag times assume a value less than or equal to the *x*-value. The measured distribution is fitted to a log-normal distribution (red dotted line) with a mean of 6.8 h and a SD of 1.3 h. A Gaussian “de-blurring” applied to these data generates the true distribution of cell lag times (blue dotted line). Both models in (A) simulate populations founded by bacteria with lag times drawn at random from this corrected distribution: cells growing independently in droplets (dashed blue) and cells dividing according to a signal from the leader cell (solid blue).

### Calculation of the uncertainty of measurement for populations founded by a single cell

*N*_*th*_ = 1.6 × 10^8^ cells ml^−1^ is the *y*-coordinate of the point taken in the exponential phase of growth to calculate the population lag time (see Fig. 1). Δ*N*_th_ is uncertainty surrounding the *y*-coordinate, i.e. derived from uncertainty on the calibration of cell concentration versus fluorescent signal (Figure S8, Supporting Information). The uncertainty of the calibration Figure S8 (Supporting Information) gives Δ*N*_th_ = 0.7 × 10^8^ cells ml^−1^ for this value as depicted by the gray area. Δ*t*_th_ is the uncertainty of the time when the population reaches beyond the threshold *N*_th_. We take its value as equal to the sampling frequency of the machine Δ*t*_th_ = 18 min. λ is the average growth rate of populations in droplets and Δλ is the uncertainty. These quantities are estimated with the distribution of the growth rate shown in Figure S6 (Supporting Information): λ = 0.84 h^−1^ and Δλ = 0.02 h^−1^. Δ*N*_0_ is the uncertainty on the inoculum size. In the experiment with populations founded by a single cell shown in Fig. 2(B), 230 − 156 = 74 droplets were empty despite being generated from the same seed culture. This is due to the randomness of the pipetting process that fills droplets of bacteria according to a Poisson process. The randomness of the process gives intrinsically an uncertainty on *N*_0_. In the following, we explain how this was estimated. Knowing the number of empty droplets allows calculation of the precise average inoculum of the experiment which correspond to the α parameter of a Poisson distribution having its first value *p*(0) = exp(−α) =74/230 : α = 1.134 cells per droplet. This average takes into account the empty droplets with zero bacteria but we only measure the nonempty droplets. To estimate the average inoculum of the nonempty droplets we draw numerically a large series of random numbers with a Poisson probability of parameter α = 1.134 and calculate the average and standard deviation (SD) of the nonzero values. This yielded an average of 1.7 and a SD of 0.9. Therefore, we consider that for our experiment in an ideal case with an infinite number of droplets the uncertainty on the inoculum of the droplets will be intrinsically Δ*N*_0_ = 0.9 cells per droplet and that the average inoculum (of the filled droplets) is *N*_0_ = 1.7 cells per droplet. All together these values allow calculation of the uncertainty of the lag time estimated by Equation (1). The expression of uncertainty is given by Equation (2) and numerical application gives Δθ = 0.88 h.

## Results

### High-throughput quantification of bacterial population dynamics with millifluidic technology

To investigate the relationship between inoculum size and duration of lag phase, we used a millifluidic device to quantify the dynamics of bacterial population growth across time (Baraban et al. 2011, Boitard et al. 2015, Cottinet et al. 2016). The device allows the monitoring of 230 bacterial populations compartmentalized in droplets contained within a tube. Figure 1(A) shows a portion of the tube with two droplets filled with cells. The statistical power of the experiment comes from precise control of large numbers of droplets, in terms of both inoculum size and homogeneity of culture conditions: Fig. 1(B) shows the growth dynamics of 40 replicate populations. Exponential growth and stationary phase are clearly seen, while lag time is concealed behind the detection threshold (gray area). Figure 1(C) shows the growth dynamics of a population contained within a single droplet.

Population density in the droplet is reported by fluorescence intensity from GFP-labeled bacteria with parameters describing the phases of growth being estimated from these time series (see Fig. 1C). Exponential growth rate, λ, is the maximum slope of the time series on a y-semi-logscale (we use a Gaussian processes method that makes no *a priori* assumption about the shape of the growth curve (Swain et al. 2016)). Final population size is estimated directly from measurements; death phase is not significant in our experiment and is ignored. Lag phase τ, is the time cells spend in a nondividing phase prior to onset of exponential growth. Hardware limitations mean that fluorescence data are unobtainable for cell densities below  1600 cells per droplet (4 × 10^6^ cells ml^−1^) and, thus τ must be estimated indirectly. This indirect measurements allows also to circumvent to classical *caveat* for lag time measurement related to variation of the fluorescence per cell during lag phase. This is done by firstly taking an arbitrary point (*t*_th_, *N*_th_) in exponential phase where cell density is *N*_th_ = 1.6 × 10^8^ cells ml^−1^. By rearranging the equation for exponential growth: $N_{th} = N_0 e^{\lambda ( t_{th} - \tau )}$, and making τ the subject

(1) \begin{eqnarray*}
\tau = t_{th} - \log (N_{th}/N_0) / \lambda .
\end{eqnarray*}

A geometrical counterpart of Equation (1) gives the population lag time as the time point at which exponential growth (line in semi-log scale) intersects the horizontal line, which depicts inoculum density (see Fig. 1C). These measurements of lag-times provide a wealth of quantitative information on the inoculum effect, as described and interpreted in the following.

### Duration of lag phase depends on the number of founding cells

In Fig. 2(A), the average lag time from three independent experiments (colors) is shown as a function of inoculum size (diamonds). Lag time decreases monotonically from  6.4 ± 1.1 h for droplets inoculated with a single cell, to 4.4 ± 0.3 h for an inoculum size of 1024 cells. The SD, represented by the error-bars in the figure, decreases monotonically and slowly with increasing inoculum size.

We also examined the dependence of other growth parameters on inoculum size. Initial experiments showed an effect on final cell density, however, this was found to be a consequence of subtle differences in glycerol concentrations arising from dilutions of frozen glycerol–saline stock cultures used to prepare founding inocula. When corrected, no effect of inoculum size on final cell density was observed. This technical, but important experimental observation is explained in Figure S2 (Supporting Information). Additionally, no effect of inoculum size on mean growth rate was detected, although the variance across droplets decreased. Details are provided in Figure S3 (Supporting Information).

What might be the basis of the decrease in mean lag time with inoculum size? There are three mutually exclusive explanations, all recognize that populations of cells are heterogeneous with regard to individual cell lag times as a consequence of innate phenotypic variability. Explanation I posits no interaction among cells, with population lag time being set by an event equivalent to a selective sweep, i.e. initiated by the cell (or cells) with the shortest cell lag time.

Explanations II and III involve interactions among cells and can be thought of in terms of two extremes of a continuum. Explanation II posits that all cells contribute equally to the production of some growth-stimulatory factor. Explanation III recognizes that population lag time could be set by the cell with the shortest lag time and whose activity triggers division of other cells. We demonstrate below that distinguishing between these alternate explanations is possible via quantitative data obtained from the millifluidic droplet device. Making this distinction requires knowledge of the lag time distribution of populations founded by single cells.

### Precise estimation of the distribution of cell lag times from inocula containing a single bacterium

To quantify the lag time of individual bacteria, 230 droplets were inoculated by—on average—a single bacterium, resulting in growth in 156 droplets (the inoculation of droplets follows a Poisson process). For each droplet, the lag time was estimated as in Fig. 1(C). The resulting distribution of lag times is shown in Fig. 2(B) (blue dots). In this case, the lag time of each population is clearly equal to that of the founding cell. We denote the single-cell lag by θ to distinguish it from τ of larger inoculum size that may be affected by cooperative effects. The heterogeneity of cell lag times is broad, ranging from ∼4 to ∼12 h, with a mean value of *m* = 6.8 h and SD σ = 1.3 h. A Shapiro–Wilk test applied on the logarithm of the data reveals the underlying distribution can be log-normal (see also the quantile-to-quantile plot shown in Figure S7, Supporting Information). Fitting a log-normal function (green dashed line in Fig. 2) yields parameters μ = 1.9 and *s* = 0.2.

Although the fit is good, there is uncertainty in the estimation of lag times due to measurement errors that propagate to the extrapolation of Equation (1). This equation expresses the dependence of lag time on parameters *t*_th_, *N*_0_, *N*_th_, and λ for droplet populations, including the special case of a population being founded by a single cell. Expanding it to a Taylor series and assuming independent variables allows the uncertainty Δθ to be calculated as

(2) \begin{eqnarray*}
\Delta \theta = \sqrt{(\Delta t_{th})^2 + \left( \frac{\Delta N_{th}}{\lambda N_{th}}\right)^2 + \left(\frac{\Delta N_0}{\lambda N_0}\right) ^2 + \left(\frac{\Delta \lambda }{\lambda ^2}\right) ^2},
\end{eqnarray*}

where Δ*t*_th_, Δ*N*_th_, Δ*N*_0_, and Δλ correspond to the uncertainty of *t*_th_, *N*_th_, *N*_0_, and λ, respectively. Given the values of these uncertainties, we estimate Δθ = 0.88 h (see “Materials and Methods” for details of calculations).

The uncertainty associated with direct measurements blurs the “true” distribution of single-cell lag times, which is less dispersed, i.e. has a smaller SD. We assume a Gaussian noise of zero mean and a SD equal to the measurement uncertainty σ_*noise*_ = Δθ. Deconvolution of the Gaussian noise from the measured distribution (Blackwood 1995) amounts to subtracting its mean and variance from that of the measurements

(3) \begin{eqnarray*}
\langle \theta \rangle = m - m_{noise} \approx 6.8 h,
\end{eqnarray*}

(4) \begin{eqnarray*}
\sigma ^2 = \sigma ^2 - \sigma _{noise}^2 \approx 1.0 h^2.
\end{eqnarray*}

The corrected distribution remains lognormal, with parameters $\mu = \log \left( \langle \theta \rangle ^2 / \sqrt{\sigma ^2+\langle \theta \rangle ^2} \right) = 1.9$ and   *$s^2 = log(\sigma^2/\langle\theta\rangle^2 + 1) = 0.14$*. Expression of the true probability density of lag time is thus

(5) \begin{eqnarray*}
f(\theta ) = \frac{1}{\theta s\sqrt{2\pi }}e^{-\frac{(\ln (\theta )-\mu )^2}{2s^2}}.
\end{eqnarray*}

The red dotted line in Fig. 2(B) depicts the corresponding CDF after correction for measurement noise; it is narrower than that obtained by direct measurement. This distribution can now be used to examine the previously proposed explanations for the dependence of population lag time on inoculum size.

### A sweep initiated by cells with the shortest lag time is inconsistent with the data

Intuitively, one might imagine that the large variability in single-cell lag-times is sufficient to account for the observed inoculum effect, even for independently growing cells: larger inocula contain outlier cells that are fast to resume growth; these could in principle take over the population and reach maximal cell number faster, causing the observed inoculum effect. Having a precise estimate of the single-cell lag time distribution, it is now possible to put this hypothesis (Explanation I) to quantitative testing.

To this end, growth of populations within droplets established from different numbers of founding cells were simulated and the match with experimental data determined. Virtual droplets were founded by cells with lag times drawn from the true distribution (shown in Fig. 2B) and with exponential growth rates drawn from the measured distribution (see Figure S6, Supporting Information). Note that addition or not of the weak correlation between lag time and growth rate seen Figure S6 (Supporting Information) does not affect the conclusion of the simulation (code provided in Appendix, Supporting Information). Cells were then allowed to replicate within droplets. To mimic the experimental protocol, the time *t*_th_ at which populations reach *N*_*th*_ = 64 000 cells (equal to a density of 1.6 × 10^8^ cells ml^−1^) was determined. Equation (1) was then used to calculate the lag time of each population with known *N*_0_ and with known mean growth rate λ. The blue dotted line in Fig. 2(A) shows the results of these simulations.

In marked contrast to the experimental results, these simulations of independent (non-interacting) cells show almost no dependence of the mean population lag time on inoculum size. In addition, the decrease in variation across droplets, represented by the SD of lag time (shaded blue region around dotted line), decreases rapidly, whereas in the experimental data the SD decreases much slower.

Failure of Explanation I to account for the data can also be understood by a simple calculation based on the growth characteristics. The corrected CDF of the single-cell lag times, Fig. 2(B), has a value of 0.025 for lag time 5 h. In other words, in a droplet inoculated by 1024 cells, ≈25 cells (0.025 × 1024) have a lag time equal to or shorter than 5 h. Similarly, the number of cells exiting lag phase between 5.8 and 7.8 h (around the mean 6.8 ± 1 h) is: (0.86 − 0.14) × 1024 ≈ 737 cells. Given the single-cell growth rate λ = 0.84 h^−1^ (Figure S6, Supporting Information), the generation time is *g* = 0.83 h. Thus, the 25 cells that start dividing before 5 h go through roughly (6.8 − 5.0)/*g* ∼ 2 generations before the 737 cells around the mean start dividing. A total of two generations of 25 cells yields 100 offspring; therefore, clearly cells with a short cell lag time do not exert a dominant sweep effect on the population.

The above estimate implies that, during the time of the measured growth, many lineages within droplets grow simultaneously and produce offspring. Therefore, if cells are independent, the population lag time is expected to be roughly equal to the mean of the single-cell lag time distribution, which is independent of inoculum size as the simulation shows. Moreover, as an average over many cells, the lag time variability across droplets should decrease as $1/\sqrt{N_0}$ with inoculum size. Indeed, the SD of the simulation shown in Fig. 2(A) decreases rapidly with inoculum size, in marked contrast to the experimental data—which decrease slowly. This discrepancy of the variance behavior with inoculum size indicates that population lag time does not arise as an average over independent cells in the inoculum. Alternative scenarios where cells are not independent are considered below.

### A leader cell triggering end of lag time for the population is consistent with the data

We now turn to test Explanations II and III that involve interactions among cells within the founding inoculum. At one extreme case (Explanation III), collective growth is governed by a single event that synchronizes population growth to the cell with the shortest lag time. This would happen if the cell that divides first signals this event to other cells, such that the entire population exits lag phase almost simultaneously. We first examine the consequences of this assumption and compare it to the data, and then consider the alternative scenario, namely, Explanation II, in which interactions among cells involve all cells contributing equally to the production of a growth-stimulating factor.

In statistical terms, we assume that an inoculum of *N*_0_ cells is a random sample from the single-cell lag time distribution *f*(θ). If there is a leader cell that triggers growth for all other cells, the measured population lag time will be equal to the shortest cell lag time in the sample, θ_*min*_. EVT provides a framework for statistical analysis of the extreme value of a sample, such as the shortest lag time θ_*min*_ among *N*_0_ cells (Embrechts et al. 2013). In the limit of large samples, EVT predicts the dependence of the mean and variance of a collection of θ_*min*_ coming from samples of size *N*_0_. It also predicts that the distribution of θ_*min*_ from populations founded by cells of a given inoculum size will approach a limiting fixed shape after appropriate normalization as the sample size increases; the precise shape is determined by global properties of the single-cell distribution *f*(θ).

The unique features of our experiment create an ensemble of droplets with controlled inoculum size, and a measurement of the population lag time for each, labeled τ. These data provide the statistical properties required to test the hypothesis that τ = θ_*min*_(*N*_0_), namely that the population lag time is equal to the minimum cell lag time among the *N*_0_ single cells of the inoculum. For this, we use predictions given by EVT on the distribution of θ_*min*_ and ask whether they are consistent with the statistical properties of τ as measured in the droplets.

The first prediction is that both the average and the SD of θ_*min*_ from populations decrease slowly with inoculum size *N*_0_. The precise scaling is derived from the single-cell distribution *f*(θ) (see Appendix, Supporting Information); for a lognormal distribution we find the scaling to be

**Figure 3.**
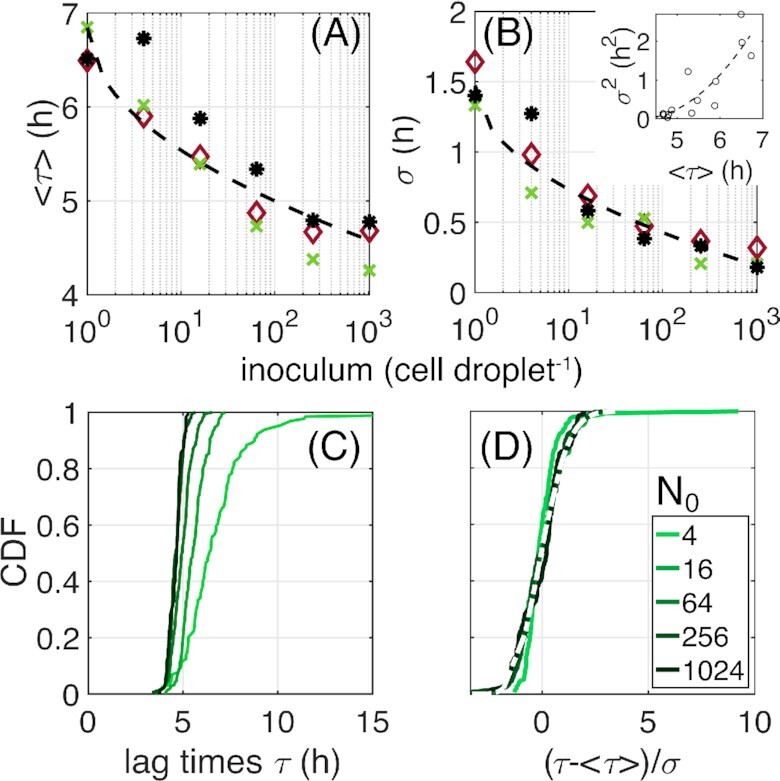
Statistical properties of lag times. For three independent experiments (colored symbols), mean lag times (A) over populations and their SD (B) are depicted as a function of inoculum size. Each point is calculated over 40 replicate populations (droplets). Inset: variance as a function of mean. The scaling relations predicted by EVT are shown in dashed lines: $y=6.84\! -\! 0.86\sqrt{\ln N_0 }$ for the mean and $y= 1/\sqrt{\ln {N_0}}$ for the SD. (C) Cumulative Distributions of population lag times for different inoculum sizes [*N*_0_, colors; legend in (D)]. The curves derive from the pooled data of three independent experiments yielding at least 120 population lag times for each. (D) Same data as in (C), scaled by subtraction of empirical mean and division by SD. The white dashed line depicts the fit by the universal distribution predicted by the EVT. The *y*-axis is shared between (C) and (D).

(6) \begin{eqnarray*}
\langle \theta _{min}\rangle \!\sim A\! -\! \sqrt{\ln N_0 },
\end{eqnarray*}

(7) \begin{eqnarray*}
\sigma (\theta _{min}) \sim B/\sqrt{\ln {N_0}},
\end{eqnarray*}

where *A* and *B* are constants. Both predictions agree well with the population lag time τ. Fitting the curve of Equation (6) to the experimental relationship between mean population lag time (〈τ〉) and inoculum size, reveals a close match (Fig. 3A). The same holds for the SD fitted to Equation (7) (Fig. 3B). We note that although testing this prediction involves fitting constants, the dependence on sample size *N*_0_ through $\sqrt{ \ln { \left( N_{0} \right)} }$ is nontrivial and unique to the predictions of EVT.

A further prediction is that the distribution of minimal values (θ_*min*_), drawn from different sample sizes, tends to a universal shape in the limit of large samples. Although, strictly speaking, this holds in the limit *N*_0_ → ∞, in practice it may be expected to hold also for finite samples—even as small as several dozen. For each sample size *N*_0_, our experiment provides a distribution of population lag times (τ), estimated over all droplets with the same inoculum size. These CDFs are depicted in Fig. 3(C) for all inoculum sizes of at least four cells. To test whether the prediction on θ_*min*_ holds for the population lag time τ, we normalize each CDF of τ by subtracting its mean and dividing its SD. Figure 3(D) shows the result of this normalization and demonstrates that the distributions of τ collapse on one another, consistent with our hypothesis τ = θ_*min*_. The lightest shaded curve, corresponding to inoculum *N*_0_ = 4, deviates from the rest—possibly indicating that this sample size is too small to acquire the limiting shape.

The universal distribution itself is also predicted by EVT (Wikipédia 2018). Its CDF has the form

(8) \begin{eqnarray*}
F(\theta _{min})=e ^{-(1+kz)^{-1/k}}, \quad z\!=\!(\theta _{min}\!-\theta _0)/\gamma ,
\end{eqnarray*}

with location and scale parameters θ_0_, γ, and a shape parameter *k* that reflects properties of the parent distribution *f*(θ), specifically the decay at its tails. Fitting the normalized data with this formula reveals an excellent match between the universal distribution formula (white dashed line in Fig. 3B) and the normalized measured distributions of τ (green lines), at least for inoculum sizes above four cells per droplet. The analytical formula for the distribution justifies the empirical procedure of normalizing by sample mean and SD used above as a test for the universal shape (see Appendix, Supporting Information).

As a corollary of the predictions in Equations (6) and (7), the variance and mean of the distributions of extreme values drawn from different sample sizes follow a well-defined relationship (see Appendix). The agreement of this relationship with the population lag time data is shown in the inset of Fig. 3(B).

Taken together, the scaling of the mean and SD of τ according to the inoculum size [Equations (6) and (7)], the resulting relationship between variance and mean of τ, the collapse of normalized distributions of τ at different sample (inoculum) sizes, and the fit of the normalized distribution to the theoretical formula Equation (8), are consistent with population lag time being equal to the shortest cell lag time in the inoculum: τ = θ_*min*_. With our understanding that growth involves multiple simultaneous lineages, we conclude that, at the time of the shortest lag time in the inoculum, many cells must start growing in parallel.

### A single leader cell determines population lag time

The agreement of statistical properties with predictions from EVT suggest that exit from lag phase is triggered by a single event—possibly a single leader cell—that signals the exit from lag to all other cells. To test this hypothesis, we performed a set of simulations where cells are not independent. As for previous simulations, at each inoculum size thousands of virtual populations were generated (see simulation code in the Appendix). For each inoculum, the cell lag time of each founder cell was drawn at random from the experimental single-cell lag time distribution (Fig. 2B) and then set to the shortest cell lag time in the sample. This means that all founder cells begin to proliferate at the same time as the leader cell with population lag time τ = θ_*min*_. As in the previous simulation, the numerical population was allowed to grow exponentially with population lag time being estimated as per the experiments.

The results are depicted by the solid blue line in Fig. 2(A) and are a close match to the experimental data over three orders of magnitude in inoculum size. Note that this is not a fit: the only input is the true single-cell lag distribution measured for populations founded by a single cell (Fig. 2B). Additionally, results from the simulations match the slow decrease of lag time variability among droplets observed in the experiments (shaded area Fig. 2A). Our results, thus provide an explanation for the relationship between size of the founding inoculum and population lag time, which are fully consistent with resumption of growth of all cells in the inoculum being triggered by a leader cell.

Thus far, there remains uncertainty as to whether population growth is triggered by a single cell, or a small group of cells. To investigate, we performed further simulations (see code in Appendix) in which cells produce a growth activator after exit from lag phase. The activator triggers end of lag phase for all cells in the inoculum upon passing a threshold. To have an effect, leader cells must activate growth of neighboring cells. The time to reach the threshold is driven by two parameters related to the activator: the concentration threshold and rate of production. The ratio of the concentration threshold over the rate of production scales with time to reach the concentration threshold. For an effect to be evident, time to reach the threshold must be less than the lag time of neighboring cells (here 6.8 h on average). Thus, to study the influence of the time to reach the threshold it is necessary to fix the production rate and vary the concentration threshold (or the other way around). In the following simulation, we chose to fix the production rate and vary the concentration threshold.

Given lack of knowledge concerning the nature of the activator, we assume that the rate of production is equal to the population growth rate. Here, the concentration threshold determines the fraction of cells in the inoculum that affects exit from lag phase. If the threshold is low, then production of growth activator by a single cell is sufficient to trigger end of lag phase for the entire population. If the threshold is high, then it is likely that multiple cells contribute to production of the growth activator. The duration of lag phase for every cell was derived from experimental data as above, and set by drawing a random value from the single-cell lag distribution Fig. 2(B).

**Figure 4.**
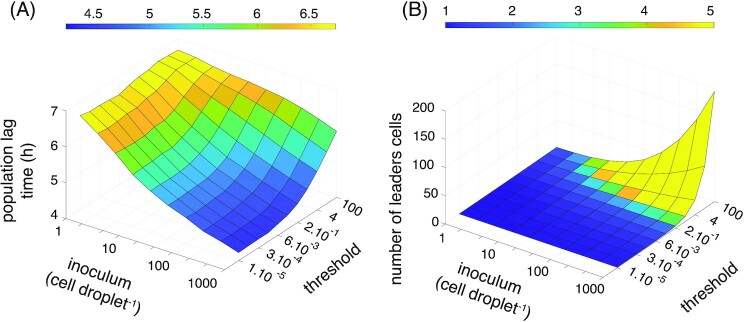
How many leader cells? Results of simulation in which each cell produces a growth activator as it exits lag phase, drawn from the experimental distribution. The activator accumulates to a critical threshold and triggers end of lag phase for the entire population. (A) Population lag time as a function of inoculum size (*x*-axis) and threshold of growth activator (*y*-axis). (B) Number of leader cells that have exited lag phase before the critical activating threshold was reached. Note that the color-bar corresponds to a narrow range of between 1 and 5 cells.

We performed simulations for a range of activator thresholds at different inoculum sizes. The results are depicted in Fig. 4. First, it is seen in Fig. 4(A) that a strong dependence of population lag time on inoculum size appears only when the threshold concentration of growth activator is low. Given the significant inoculum effect observed in our experiments, we conclude that this result is consistent with our data only in the region of a low activation threshold. Second, Fig. 4(B) shows the effective number of cells that “lead” the population to exit from lag phase. This number is defined by those cells, which had already reached the end of their individual lag time as drawn from the single-cell distribution, before the threshold was reached. Strikingly, we see that for a large range of activator concentrations, only a single cell has time to exit lag phase before the critical concentration of activator is reached. These simulations support the conclusion that our experimental observations are consistent with Explanation III, where a single leader cell ends lag phase for the entire population.

## Discussion

An inverse relationship between the number of bacterial cells founding a new environment and the time to exit lag phase was first noted more than 100 years ago (Rahn 1906, Penfold 1914). Despite its significance, rigorous validation has been lacking, and understanding of the causes and controlling factors remains incomplete. Paucity of progress has stemmed largely from difficulties associated with experimental analysis of populations founded by few cells.

Here, taking advantage of new opportunities presented by millifluidic technologies (Baraban et al. 2011, Boitard et al. 2015, Cottinet et al. 2016) we have obtained quantitative evidence from highly replicated populations founded by controlled numbers of cells, that in populations of *P. fluorescens* SBW25, the time to resume growth after transfer to a new environment is strongly influenced by size of the founding inoculum. Moreover, the same technology has allowed determination of the duration of lag phase for a sample of individual cells. The average decrease in time to growth resumption, variance across droplets, and distribution shapes, follow predictions of EVT, consistent with the inoculum lag-time being determined by the minimum value sampled from the single-cell distribution. At the same time, within droplets, growth of multiple cell lineages in parallel contribute to population expansion with no single lineage providing a disproportionate effect on duration of lag time. Our experimental results, thus show that exit from lag phase depends on strong interactions among cells, suggesting that a “leader cell” triggers end of lag phase for the entire population.

This finding builds on recent work in which the time to first division of single bacteria maintained in isolated cavities of microfluidic devices has been measured (Julou et al. 2020, Moreno-Gámez et al. 2020, Şimşek and Kim 2019). From such studies, it is clear that there is substantial variation in cell-level lag time with evidence that this variance can have profound fitness consequences for population growth. For example, in fluctuating environments, heterogeneity in the time for individual cells to resume growth, can facilitate survival in the face of environmental change (Julou et al. 2020), especially that wrought by periodic antibiotic stress (Fridman et al. 2014, Şimşek and Kim 2019, Moreno-Gámez et al. 2020).

Although microfluidic chambers used for analysis of isolated cells allow precision measures of the distribution of lag times for single cells, such experimental devices do not allow for interactions among cells, thus making problematic any attempt to connect the distribution of single-cell lag times to population lag times. In fact, extrapolation of population lag times from knowledge of the distribution of single-cell lag times would be justified only in the case of independent cells.

Linking cell and population level behaviors necessarily requires measures of lag times both for individual cells and for populations in precisely the same environment. Moreover, the environment should be well-mixed (spatially homogeneous and devoid of surface effects) so that should emergent population-level behaviors be relevant, mediated via, e.g. production of diffusible growth factors (Lankford et al. 1966, Kaprelyants and Kell 1996), then effects can be observed. In this regard, the millifluidic device has proven fit for purpose.

In seeking an explanation for the observed inoculum effect, we considered three mutually exclusive explanations. Central to Explanation I was absence of interactions among cells with the inoculum effect being explained by disproportionate growth of a subset of cells with the shortest time to first cell division. Both simulations and simple calculations led to unequivocal rejection of this hypothesis.

Explanations II and III recognized the possibility of interactions among cells. Because of the power of EVT, combined with well-understood statistical properties, we chose to focus on whether population lag times were determined by the minimum value sampled from the single-cell distribution (Explanation III). EVT makes predictions as to the distribution of minimal cell lag times across droplets, which surprisingly, hold for the distribution of population lag times measured in experiments, leading to the conclusion that population lag time is equal to the minimal cell lag time present in the inoculum. Simulations of population growth in droplets based on this evidence delivered an almost perfect match to experimental data. While conformity to Explanation III means that Explanation II in a strict sense (in which all cells contribute equally to exit from lag phase) cannot hold, the fact that there is a continuum of possibilities led us to perform additional simulations to address whether our data are consistent with resumption of growth being triggered by just a single leader cell. In these simulations, a growth activator was assumed to be produced by all cells as they exited lag phase, but was required to accumulate to a threshold before all other cells started growing; this interpolates between a single leader cell and a contribution from all cells, depending on the threshold level. We found that a strong decrease of population lag with increasing inoculum size was reproduced in simulations, but only when the threshold was low. In the relevant parameter region where the inoculum effect matched the experiment, we found that only a single cell contributes to the production of growth activator.

In our simulations, it was assumed that droplets are well-mixed (see also Figure S13, Supporting Information) so that the time for transport of growth activating molecules is negligible. In reality, three times scale are relevant: the time it takes for single cells to exit lag phase (reported in Fig. 2B at 6.8 h on average), the rate of production of the growth activating molecule, and the time it takes for the activator to propagate through the population. Transport is dominated either by diffusion or convection, with the former generally slower than the latter. In our droplet-based system, continual movement of droplets (and thus stirring of contents) points to convection as the primary mechanism of signal propagation; thus, as soon as the signal is produced, it is likely to spread in the droplet and be sensed by all other cells. Convection is relevant in many cases, including stirred reactors, moisture droplets on food, local environments within eukaryotic hosts, moving water bodies, and even undisturbed culture flasks (Ardré et al . 2019). In the absence of convection—and in environments where the time to transport effector molecules is longer than the intrinsic lag time of cells—the inoculum effect may be of less significance.

The rate at which the signaling molecule is produced is a key factor especially in environments of large volume. For instance, if the rate of production is low, then the leader cell will fail to produce sufficient signaling molecule to trigger neighboring cells. Under such circumstances, no inoculum effect will be observed and resumption of population growth will be determined by the intrinsic lag time of each cell. In our simulations (Fig. 4), we assumed that the production rate is equal to the population growth rate (Figure S3, Supporting Information), which while a reasonable assumption, lacks, at the present time, a mechanistic basis.

An obvious next question concerns identity of the growth activator. While detailed investigations are beyond the scope of this study, we nonetheless, considered the possibility that iron chelation might trigger the effect. Such a possibility has been previously suggested (Kaprelyants and Kell 1996). To this end, we repeated the initial experiment in which the time to resumption of growth was determined in replicate populations founded by different numbers of cells as in Fig. 2(A). Instead of SBW25, a mutant deficient in production of the iron-chelating compound pyoverdin was used: *P. fluorescens* SBW25 *pvdS*G229A (Zhang and Rainey 2013). No change in the inoculum effect was observed (see Figure S4, Supporting Information), thus ruling out pyoverdin as the activating molecule. We next asked whether the inoculum effect might be eliminated by addition of culture supernatant, derived from an overnight culture of SBW25 grown in CAA, to populations of cells in droplets. The ensuing data show that indeed culture supernatant significantly dampens the inoculum effect (Figure S11, Supporting Information). This is consistent with Lankford et al. (1966) and Kaprelyants and Kell (1996), who observed an inoculum effect in flasks that could be abolished by addition of culture supernatant.

A further set of factors that stand to influence the inoculum effect, stem from the environment. The effect of differences in chemical composition of growth media, growth stage of cells, and external stressors are presently unknown, but the subject of current investigation.

The relationship between the number of cells founding growth in a new environment and duration of lag phase has profound implications for microbiology. While much remains to be understood, including generality and molecular bases, the rigorous quantification achieved here provides unequivocal evidence of an inoculum effect in *P. fluorescens* SBW25. Moreover, we show that the effect is best understood in terms of interactions among cells with statistical analysis of the distribution of population lag times indicating that a single leader cell is sufficient to trigger simultaneous growth of all cells in the founding population.

## Supplementary Material

uqac022_Supplemental_File
