# Correction to: A leader cell triggers end of lag phase in populations of Pseudomonas *fluorescens*

**DOI:** 10.1093/femsml/uqad040

**Published:** 2023-10-20

This is a correction to: Maxime Ardré, Guilhem Doulcier, Naama Brenner, Paul B Rainey, A leader cell triggers end of lag phase in populations of Pseudomonas fluorescens, microLife, Volume 3, 2022, uqac022, https://doi.org/10.1093/femsml/uqac022

In the originally published version of this manuscript, there was an error in the following sentence in the Methods section:

CAA for 1 l: 5g of Bacto Casamino Acids Technical (BD ref 223120), 0.25 g MgSO_4_·7H_2_O (Sigma CAS 10034-99-8), and 0.9 g K_2_HPO_4_ (Melford CAS 7758-11-4).

The sentence has been corrected to read as follows:

CAA for 1 l: 5g of Bacto Casamino Acids Technical (BD ref 223120), 0.25 g MgSO_4_·7H_2_O (Sigma CAS 10034-99-8), and 0.9 g K_2_HPO_4_ (Sigma CAS 7758-11-4).

