# Supplementary material for: A leader cell triggers end of lag phase in populations of Pseudomonas fluorescens
Source: Microlife. 2022 Nov 2;3:uqac022. doi: 10.1093/femsml/uqac022 (PMC10117806; doi:10.1093/femsml/uqac022)
Supplement: uqac022_Supplemental_File [file uqac022_supplemental_file.pdf]

Supplementary Appendix

Statistical properties for extreme values

EVT states that the minima of finite samples, drawn from some parent distribution, exhibit predictable statistical properties in the limit of large sample sizes (Embrechts et al., 2013). These properties are testable experimentally in our system, where we can control the sample size - inoculum size N_0, and repeat the sampling many times. The theory predicts how the mean and variance of sample minima depend on N_0, given the parent distribution. It moreover contains the powerful statement that the distribution shape converges to a universal one in the limit $N_0 \to \infty$, which reflects the decay of the original distribution tails. Similar statements hold for maximum as well as other extreme values (e.g., second largest, etc).

To develop some intuition for the scaling of moments with sample size, imagine drawing N_0 random variables from a normal Gaussian distribution. To estimate the minimal drawn number, we divide the real line into N_0 equal-probability bins (see Fig. 5). On average, there will be one number drawn from each bin; therefore we may estimate that the minimal value lies within the lowest bin, in the range $(-\infty, x_1)$. Clearly, the larger the sample, the more bins we can use and still have an average of one number in the lower one; as the sample becomes large, we are increasing our chance of sampling low-probability events in the tail of the distribution.

To obtain the qualitative nature of the scaling, we seek a relation between the sample size N_0, also the number of bins, and the values of the continuous variables x in the lowest bin. Comparing the probabilities,

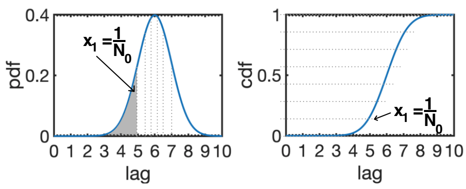

Fig. 5. Drawing a finite sample of size N_0 from a continuous Gaussian distribution. To estimate the minimal value drawn out of N_0 values, we divide the real line into N_0 equi-probable bins; in the figure, $N_0 = 7$. The estimate that the minimum resides in the lowest bin can be used to derive the scaling $\sqrt{\ln N_0}$ in the limit of large N_0. Left we represent the probability density function of a Gaussian (mean 6h, SD 1h). The probability to have value in the lowest bin between 0 and $x_1 = \frac{1}{N_0}$ is equal to the area of this bin highlighted in grey. Right we represent the cumulative density function of the same Gaussian. The probability to have a value below or equal to $x_1 = \frac{1}{N_0}$ is equal to the y-value of the lower dotted line pointed out by the arrow.

$$\frac{1}{N_0} = \frac{1}{\sqrt{2\pi}} \int_{-\infty}^{x_1} e^{-x^2/2} dx. \quad (9)$$

For large N_0, x_1 is very small and the integral is dominated by the uppermost limit. This means that if a random number is drawn from the lower bin, it is highly likely equal (or close to) the upper end of the bins; the chance of getting other values inside the bin are exponentially smaller. Therefore we approximate

$$\int_{-\infty}^{x_1} e^{-x^2/2} dx \approx e^{-x_1^2/2}.$$

With this relation, we can solve for $x_1(N_0)$ in Eq. 9 to find

$$x_1 \sim \sqrt{\ln(N_0)}.$$

This value of x_1 gives the leading scaling behavior of the minimal random number out of a large sample N_0, on average. It is a rough estimate and can be done more rigorously by utilizing the asymptotic expansion of the error function rather than the end value alone.

Although this argument is made for Gaussian random variables, the same scaling holds also for the lognormal ones. Additionally, it was made for a zero-mean and unit-SD distribution; to account for arbitrary mean and variance of the parent distribution, in Fig. 3 we shift and scale by the nonzero mean and non-unit SD, respectively.

Empirical testing of scaling property

A unique feature of our experiment is the measurement of population lag time distributions for different controlled inoculum size. Thus, we can test whether these distributions have a shape that is predicted by the EVT because the population lag times would be actually equal to a minimal cell lag time. In the following we show that all the distributions collapse on one another after appropriate scaling indicating that they have indeed a common shape underpinned by EVT. The analysis we propose can be done without knowing the parameters of the distribution and is a more robust approach compared to analysis made by using a fit which can be sensitive.

The most general form of the extreme value distribution was specified in Eq. 8 where θ_0 is the location parameter, γ the scale parameter and k the shape parameter. The mean and variance of this distribution generally depend on these parameters. In particular, they are sensitive to k; for $k = 0$ the extreme value distribution is a Gumbel distribution and for $k \neq 0$ it is a Frechet or Weibull distribution. Taking a practical perspective, we show below that it is not required to fit the parameters of the distribution in order to test for their scaling property; it is sufficient to empirically subtract the average and divide by the standard deviation.

For all cases where the first two moments exist, they are

$$\langle \theta_{min} \rangle = \begin{cases} \theta_0 + \frac{\gamma}{k}\left[\Gamma(1-k) - 1\right] & k \neq 0\,, \\ \theta_0 + \gamma\epsilon & k = 0 \end{cases}$$

$$\sigma^2(\theta_{min}) = \begin{cases} \frac{\gamma^2}{k^2}\left[\Gamma(1-2k) - \Gamma(1-k)^2\right] & k \neq 0\,, \\ \gamma^2 \frac{\pi^2}{6} & k = 0. \end{cases}$$

Here ϵ is the Euler–Mascheroni constant and Γ the gamma function. Although these are cumbersome expressions, they have the simple form

$$\langle \theta_{min} \rangle = \theta_0 + \gamma f_1(k)$$

$$\sigma^2(\theta_{min}) = \gamma^2 f_2^2(k).$$

Therefore, the two-parameter scaling by the two first moments amounts to an affine transformation of the random variable:

$$z = \frac{\theta_{min} - \langle \theta_{min} \rangle}{\sigma(\theta_{min})} = \frac{\theta_{min} - [\theta_0 + \gamma f_1(k)]}{\gamma\sqrt{f_2(k)}} = a\theta_{min} + b$$

with a, b constants that can depend on k. We show in the section below that if a variable is distributed according to a Generalized

Extreme Value distribution (GEV) with shape parameter k, $\theta_{min} \sim GEV(\theta_0, \gamma, k)$, then affine-transformed variables $z = a \cdot \theta_{min} + b$ are also GEV-distributed, with modified scale and shift parameters but with the same shape parameter: $a \cdot \theta_{min} + b \sim GEV(\tilde{\theta}_0, \tilde{\gamma}, k)$. This means that the GEV distribution shape is invariant under affine transformations. Therefore, the distribution collapse of sampled data after empirical scaling by mean and SD provides a test for their common shape, and thus for their consistency with the extreme value properties.

Using this general expressions for mean and variance, we may express $\sigma(\theta_{min})$ as a function of $\langle \theta_{min} \rangle$

$$\sigma^2(\theta_{min}) = \frac{f_2(k)}{f_1(k)^2} \left(\langle \theta_{min} \rangle - \theta_0 \right)^2 . \tag{10}$$

This relation was also tested in the measured data, as is depicted in the inset of the Fig. 3B.

Invariance of GEV under affine transformation

We here show the invariance of distribution shape under affine transformation, for the entire GEV family of distributions. Suppose $x \sim GEV(x_0, \gamma, k)$ where x_0 is the centering parameter, γ the scaling parameter and k the shape parameter. The cumulative form of the GEV is given by:

$$F_X(x) = \exp \left[- \left(1 + \frac{k}{\gamma}(x - x_0) \right)^{-\frac{1}{k}} \right]$$

Now consider the variable $y = ax + b$: the cumulative form of the distribution of y ($F_Y(y)$) can be derived from the distribution of x ($F_X(x)$): $F_Y(y) = \text{Prob}\,(Y \leqslant y) = \text{Prob}\,(aX + b \leqslant y) = \text{Prob}\left(X \leqslant \frac{y-b}{a} \right) = F_X\left(\frac{y-b}{a} \right)$. Therefore using the expression of F_X we can find F_Y. Using $y_0 = a x_0 + b$,

$$\begin{aligned} F_Y(x(y)) &= \exp \left[- \left(1 + \frac{k}{\gamma} (\frac{y-b}{a} - x_0) \right)^{-\frac{1}{k}} \right] \\ &= \exp \left[- \left(1 + \frac{k}{\tilde{\gamma}}(y - y_0) \right)^{-\frac{1}{k}} \right] \end{aligned}$$

where $\tilde{\gamma} = a\gamma$. This shows that y is also distributed according to a GEV distribution, with modified shift and scale parameters but with the same shape parameter k.

It can be seen directly from the general expression of the mean and variance of the GEV family, that the scaled variable

$$z = \frac{x - \langle x \rangle}{\sigma(x)} = \frac{x}{\gamma f_2(k)} - \frac{x_0 + \gamma f_1(k)}{\gamma f_2(k)} \sim \text{GEV}\left(\frac{f_1(k)}{f_2(k)}, \frac{1}{f_2(k)}, k \right).$$

This GEV distribution of z has a mean $\langle z \rangle = 0$, a standard deviation $\sigma(x) = 1$ and only a single parameter k. This property of extreme values distribution is used in the main text Fig. 3D to demonstrate that the population lag times follows the EVT and therefore that the population lag time is equal to the leader cell lag time.

Supplementary figures

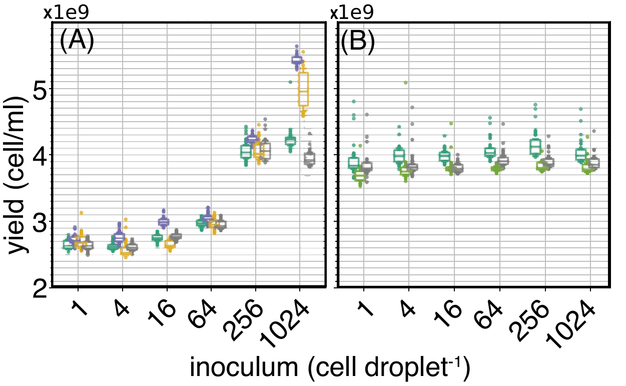

Fig. 6. Maximal density of bacteria in droplets depends on the environment. We measured the maximal density of bacteria reached for 6 different inoculum sizes (1, 4, 16, 64, 256, 1,024 cells) in rich medium. Each box plot is calculated from 40 droplets (30 for the inoculum 1,024). The different colours correspond to independent replicates. **(A)** Maximal density in droplets prepared from a 100µl glycerol stock with a "naive" serial dilution that does not compensate the glycerol concentration across bacterial inocula. We observe that the maximal density of bacteria depends on the inoculum. At high dilution, (small inoculum size), the maximal density reaches $2.6 \cdot 10^9$ cells ml^{-1} whereas at low dilution, it goes above $4 \cdot 10^9$ cells ml^{-1}. (A) and (B) share the same y-axis. **(B)** Maximal density of an "aware" serial dilution such that the concentration of glycerol is kept constant across inocula. Glycerol concentration is balanced by addition of an appropriate volume of glycerol (stock at 60% v/v) to the inocula. We see that balancing the glycerol in the droplets results in a constant maximal density of bacteria, whatever the inoculum of bacteria in the droplets. Thus, traces of glycerol coming from the frozen glycerol stocks influence the maximal density of bacteria. Diluting "naively" the glycerol of the frozen stock by 70x (together with the cells) yields an increase of 150% of the maximal bacterial density reached in droplets compared to a dilution of 70,000x. Thus, the millifluidic technology has the sensitivity to measure precisely such an abiotic effect. In our work we always took care to balance the glycerol concentration in the culture to keep it constant across inocula.

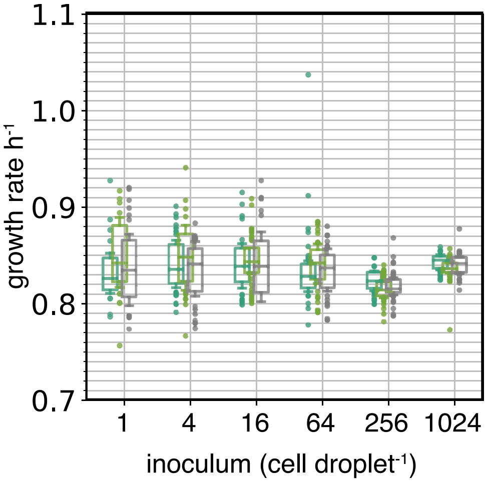

Fig. 7. Growth rate in droplets does not depend on inoculum size. Boxplots of growth rate as a function of inoculum size estimated from time-series of 40 droplets (except 1,024 which is 30). The data are the same as in Fig. 2A that shows the lag time of these droplets. The mean growth rate is approximately constant, with a median at $0.84 \pm 0.02\ h^{-1}$. The variance in growth rate decreases with inoculum size. The colours correspond to three independent experiments.

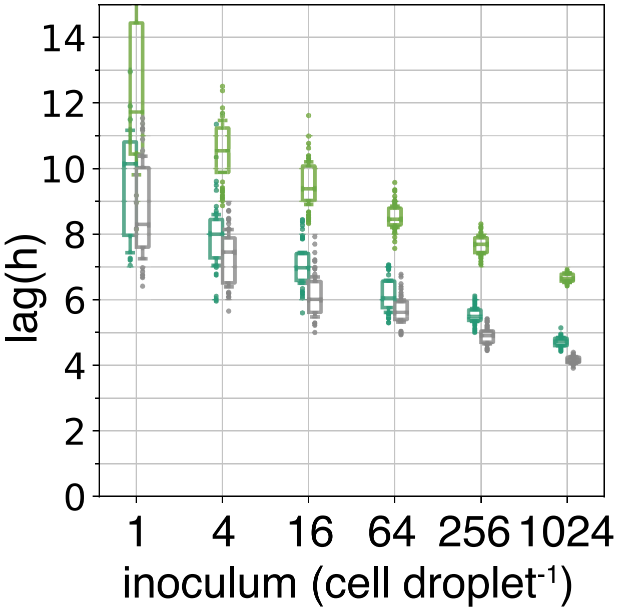

Fig. 8. Inoculum effect on the lag for *P. fluorescens* SBW25 PvdS229. *P. fluorescens* SBW25 PvdS229 does not produce pyoverdin due to a mutation in *pvdS*, a gene that encodes the extracytoplasmic family sigma factor *PvdS*, and which directs expression of the pyoverdin biosynthetic genes (Cunliffe et al. 1995). Pyoverdin is an iron chelator that allows pseudomonads to forage iron in their environment. The presence of the inoculum effect despite the non-production of pyoverdin indicates that this metabolite does not play a role in the coordinated exit of lag phase. The three colours correspond to three independent experiments in fresh CAA. Data are represented in boxplot for every inoculum (with jitter for ease of visualisation)

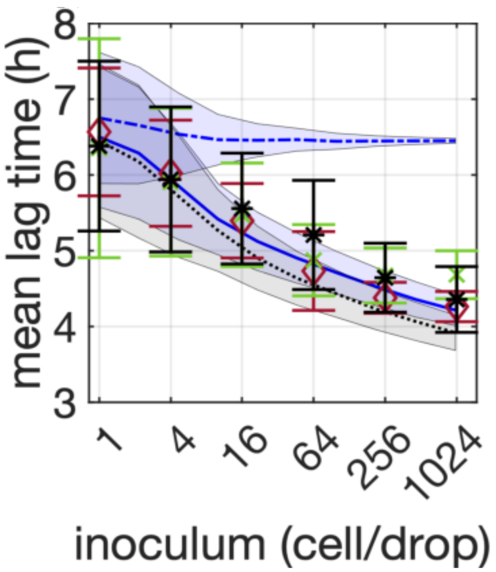

Fig. 9. Effect of deconvolving the measurement error on the single-cell lag time distribution. The same data (symbols) and simulation (solid blue line) shown in Fig. 2A are shown. In addition, results of the simulation where no deconvolution is performed on the cell lag time distribution (Fig. 3B) is shown in black dotted line. This simulation uses directly the raw cell lag time. It under-estimates significantly the mean lag time as a function of inoculum size.

Fig. 10. Statistical properties of populations founded by a single cell. The left panel shows the probability density function of the growth rate in the experiment with populations founded by a single cell (Fig. 3B) plotted with the Matlab© command: *histogram(data,'Normalization', 'pdf')*.

. Growth rate is defined as the maximum value of the derivative of the growth curves (Fig. 1C). Estimation of the maximal value of the derivative for a droplet is given with the SD (shaded area around the derivative Fig. 1C). The inset reports the histogram of the SDs. The mean value of the SDs is taken as the typical error of the growth rate $\triangle\lambda = 0.02$. The right panel shows the correlation between lag time and growth rate in each droplet; Pearson coefficient of 0.43. Every points corresponds to a single droplet.

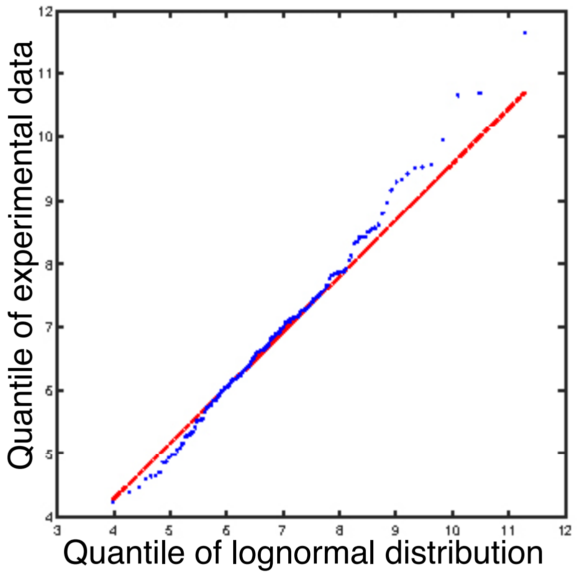

Fig. 11. Quantile to quantile plot (qqplot) of cell lag time. To determine the distribution that underpins the cell lag time (θ), we plot the quantile of a log-normal distribution versus the quantile of the distribution of the experimental measurements. The resulting quantile to quantile plot is well fitted by a line of slope 1 indicating that the experimental values are consistent with a log-normal distribution.

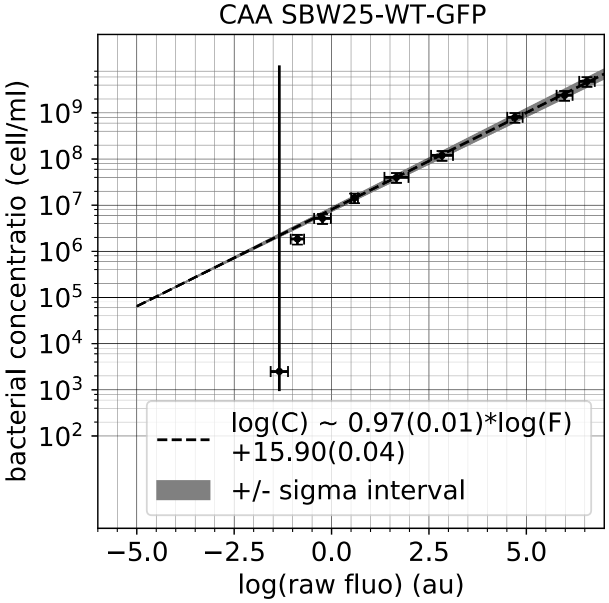

Fig. 12. Calibration curve of the voltage given by the fluorescent detector in the channel GFP to bacterial concentration. The relationship between fluorescence (raw fluo) and bacterial cell number (cells ml^{-1}). Each points corresponds to 10 droplets in the millifluidic device at a given bacterial concentration. The range of bacterial concentrations is achieved by diluting a seed culture grown over-night in CAA. The SD of the "raw fluo" across the 10 droplets is used as x-error bar. The bacterial concentration of the seed culture is measured with the standard protocol of serial dilution followed by counts of colonies on agar plates. The agar-plate count is made with 10 replications allowing estimation of SD of the seed culture counts. This SD is used in the plot as the y-error bar. The dashed line depicts a linear fit on the experimental points arranged on a log-log scale. Its equation is given in the inset with uncertainty. The vertical line depicts the value of the measured raw fluorescence for pure CAA medium (the blank). To guide the eye, the point on this line, for which the x-coordinate is the measure of raw fluorescence for droplets containing pure CAA (0 cell ml^{-1}), is placed at a y-coordinate corresponding to concentration of 1 cell droplet^{-1} (*ie* $2.5 \cdot 10^3$ cell ml^{-1}).

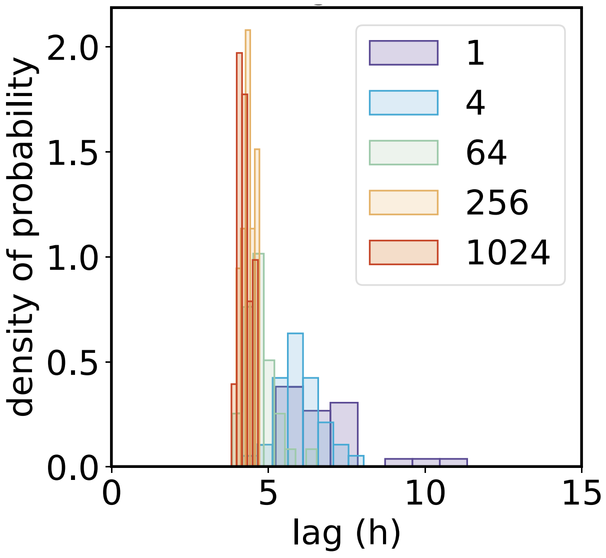

Fig. 13. Histograms of lag time for a range of inoculum sizes. These data are the same as one replicate shown Fig. 3A but here the binned histograms are displayed instead of points. The colours of the histograms in legend indicate the corresponding inoculum N_0 (in cells droplet^{-1}).

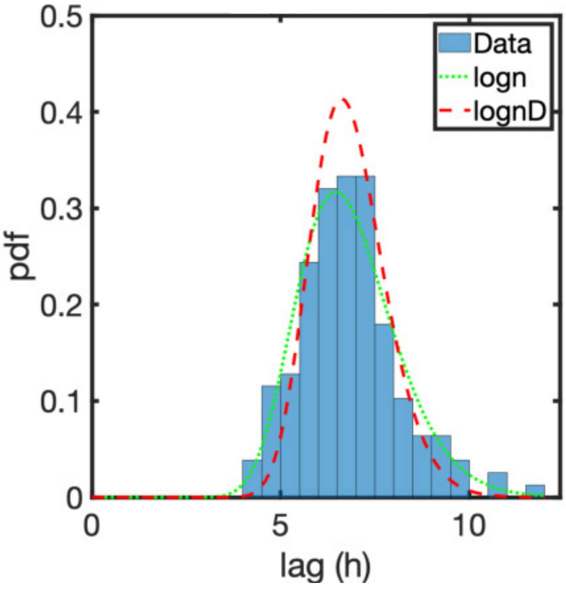

Fig. 14. Probability density function estimates of the log-normal distribution of lag time. These data are as in Fig. 3B but differently represented. The "logn" (green dotted line) is the fit of the data (blue bars) to a lognormal distribution. The "lognD" is the true log-normal distribution of lag after deconvolution of the gaussian noice.

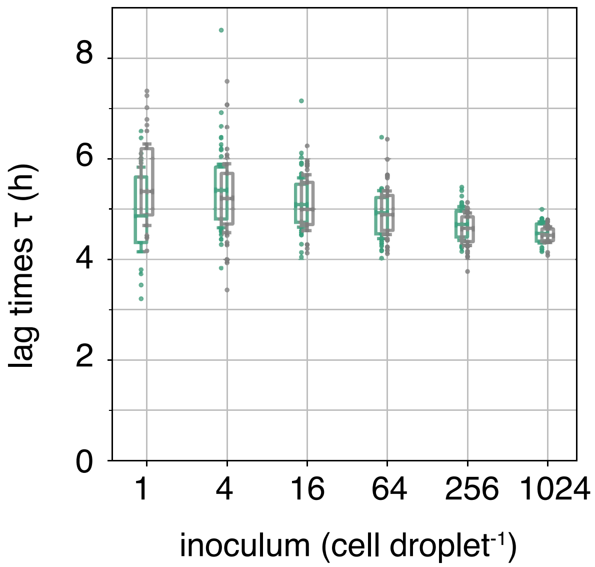

Fig. 15. Effect of supernatant on the inoculum effect. Addition of 50 µl of filtered supernatant from an overnight culture of *Pseudomonas fluoresens* SBW25 grown in CAA medium (added to 4 ml of CAA), significantly decreases the lag time of cultures started with few cells. Compared to the culture without supernatant, Fig. 2A, the lag time decreases below 6 h for all inoculum sizes and the dependence of the lag time on inoculum size becomes very weak. It is worth noting that the supernatant was ventilated several hours in a tube open to the ambient atmosphere: dissolved gases, including carbon dioxide, are therefore likely at equilibrium. The colors of the box plot indicate two independent experiment.

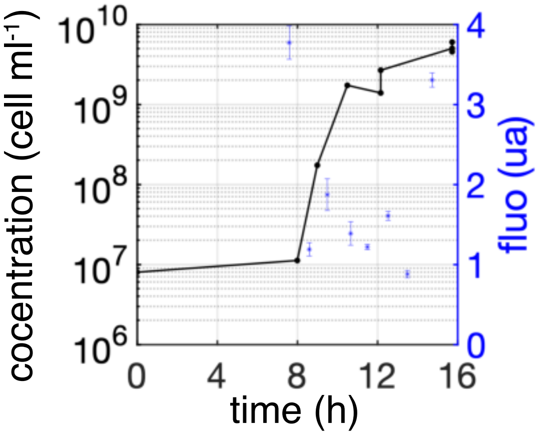

Fig. 16. Total fluorescence per cell remains constant throughout the growth phase. We measured the total GFP fluorescence per cell for SBW25 grown in rich medium (LB) with a microscope (100X). The black line reports growth of the culture with time (left y-axis) measured by CFU by counting colonies on agar plates. The right blue dots report the total fluorescence per cell at different time points (right y-axis). Every dot shows the average fluorescence for at least 1000 cells and the error bars are plus and minus the standard deviation. We do not see any trend of fluorescence per cell with growth. We conclude that the total fluorescence per cell is constant during population growth. Therefore the total fluorescence per droplet is a good proxy to measure the cell concentration per droplet.

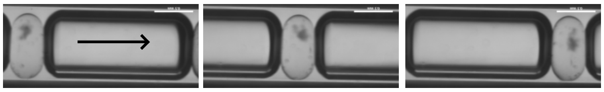

Fig. 17. Mixing of droplet contents. Droplets move back and forth. While moving the contents are well mixed. The droplet shown contains aggregating cells of *E. coli*. The drop moves left to right in the tube (depicted by the arrow). Aggregates in the drop make a circular movement. Image provided by courtesy of Millidrop©.

Codes of simulation

All the codes can be downloaded here: https://github.com/mxardre/inoculum-effect.git. We also give the code of the simulation running in Matlab©to generate the Figs. 2A and 4:

```
close all
%clear all
Vdrop = 4.4e-4; %ml

Ndrop=1000;
inocula = logspace(0,3,10); %range of inocula to simulate
dt = 1/60;
timeSpan = 0:dt:30;

Nthresh = 1.6e8; %cell/ml. Threshold to calculate the lag time like in the experiments.

SDNoise = 0.88; %SD of the noise

MM = 6.8246; %mean value of the Exp distribution of lag for logn
VV = 1.3322^2 - SDNoise^2; %SD of the Exp distribution of lag for logn
%calculate the new param of the logn to remove the noise
mu = log(MM^2 / sqrt(MM^2 + VV));
s = sqrt(log(VV/MM^2 + 1));


mg = 0.8430; %average growth rate %1/h from lag times distribution of inoculum 1 Fig. 10
vg = 0.02; %SD growth rate from lag times distribution of inoculum 1 Fig. 10

%% simulation of the exponential growth of bacteria in the droplets. Measure of the lag for each droplet.

tmes = zeros(Ndrop,length(inocula));

clear timeSeries tmes
for inoc = 1:length(inocula) %loop over the inoculum
    for i = 1: Ndrop %loop over the drop

        r = round(poissrnd(inocula(inoc))); %draw a random inoculum size according to the poisson distribution.

        timeSeries = ones(r,length(timeSpan))*nan; %time serie of the growth for each bacterial lineage in this droplet
        clear tlag
        tlag = lognrnd(mu,s,r,1); %draw randomly the cell lag according to the corrected cell lag distribution measured Fig. 2A.

        grate = normrnd(mg,vg,r,1); %draw randomly the cell growth rate according to the growth rate distribution measured Fig. 10.

        timeSeries(:,:)= exp(grate.*(timeSpan-tlag)); % proceed to the exponential growth of every bacterial lineage in this
              droplet.
        timeSeries(timeSeries<1)=1; % the lineage timeseries in the droplet must start at 1 before the division of the bacteria

        %calculation of the cell concentration in this droplet along the
        %time by summing the lineage time serie of bacteria
        l = size(timeSeries);
        if l(1)==1
            totDrop = timeSeries/Vdrop;
        else
            totDrop = nansum(timeSeries)/Vdrop;
        end

        %measure the lag of the droplet by finding the time at which the
        %cell concentration gets above Nth like in the experiments
        if r~=0
            tau = timeSpan(find(totDrop>Nthresh,1,'first'));
            if isempty(tau)
                tmes(i,inoc) = nan;
            else
                tmes(i,inoc) = ...
                timeSpan(find(totDrop>Nthresh,1,'first'))...
                -log(Nthresh*Vdrop/r)/nanmean(grate);
```

```
            end
        else
            tmes(i,inoc) = nan;
        end
    end
end

for i = 1:length(inocula)
    tstat(i) = nanmean(tmes(:,i));
    tstsdstat(i) = nanSD(tmes(:,i));
end

%% synchronistation demonstration of the synchronisation effect
clear tmes tsync tstsdsync
tmes = zeros(Ndrop,length(inocula));
for inoc = 1:length(inocula)

    for i = 1: Ndrop

        r = round(poissrnd(inocula(inoc))); %draw a random inoculum according to the poisson distribution.

        if r~=0
            timeSeries = ones(r,length(timeSpan))*nan; %timeSerie of the growth for each bacteria in this drop

            clear tlag
            %log-normal
            tlag = lognrnd(mu,s,r,1); %draw randomly the cell lag according to the corrected cell lag distribution measured Fig; 2A.

            tlag = ones(r,1) .* (min(tlag)); %set all the cell lags to the minimal cell lag of the leader

            grate = normrnd(mg,vg,r,1); %draw randomly the cell growth rate according to the growth rate distribution measured Fig. 10.


            timeSeries(:,:)= exp(grate.*(timeSpan-tlag)); % proceed to the exponential growth of every bacterial lineage in this droplet.
            timeSeries(timeSeries<1)=1; % the lineage timeseries must start at 1 before the division of its 1st bacteria.

            %calculation of the cell concentration in this droplet along the
            %time by summing the lineage time serie of bacteria
            l = size(timeSeries);
            if l(1)==1
                totDrop = timeSeries/Vdrop;
            else
                totDrop = nansum(timeSeries)/Vdrop;
            end

            %measure the lag of the droplet by finding the time at which the
            %cell concentration gets above Nth like in the experiments
            tau = timeSpan(find(totDrop>Nthresh,1,'first'));
            if isempty(tau)
                tmes(i,inoc) = nan;
            else
                tmes(i,inoc) = ...
                timeSpan(find(totDrop>Nthresh,1,'first'))...
                -log(Nthresh*Vdrop/r)/nanmean(grate);
            end
        else
            tmes(i,inoc) = nan;
        end
    end
end

for i = 1:length(inocula)
    tsync(i) = nanmean(tmes(:,i));
    tSDsync(i) = nanSD(tmes(:,i));
end
```

```
%% plot the curves of stat effect vs sync effect. Require to run the code above first.

%experimental points
lag = transpose([ 6.564471 6.018853 5.392048 4.729353 4.375776 4.259628; ...
    6.349308 5.901676 5.470768 4.871843 4.669578 4.680528; ...
    6.377465 5.937269 5.553422 5.204688 4.640315 4.353197]);

slag = transpose([...
    0.843766 0.700523 0.491303 0.519735 0.203902 0.199777;...
    1.445141 0.974685 0.682811 0.469918 0.363288 0.316424; ...
    1.120196 0.956412 0.731424 0.720511 0.455629 0.434152 ]);

N0 = transpose(repmat([1 4 16 64 256 1024], size(lag,2), 1));


%plot the figure

figure('Renderer', 'painters', 'Position', [10 10 900 900]),
hold on

alpha = 0.1;
y = tsync; % your mean vector;
x = log(inocula);
SD_dev = tSDsync;
curve1 = y + SD_dev;
curve2 = y - SD_dev;
x2 = [x, fliplr(x)];
inBetween = [curve1, fliplr(curve2)];
fill(x2, inBetween, 'b','FaceAlpha',alpha);
plot(x, y, '.-b', 'LineWidth', 5,'MarkerSize',20,'DisplayName', 'Leader');

y = tstat; % your mean vector;
x = log(inocula);
SD_dev = tstsdstat;
curve1 = y + SD_dev;
curve2 = y - SD_dev;
x2 = [x, fliplr(x)];
inBetween = [curve1, fliplr(curve2)];
fill(x2, inBetween, 'b','FaceAlpha',alpha);
plot(x, y, '.-.b', 'LineWidth', 5,'MarkerSize',20,'DisplayName', 'Stat');

clr = {'b' 'r' 'g' 'k'};
for i = 1:size(lag,2)
    h = errorbar(log(N0(:,i)), lag(:,i), slag(:,i),'d', 'MarkerSize',20, 'color', clr{i+1}, 'LineWidth',3, 'CapSize', 40);
end

set(gca,'FontName','Helvetica')
xlim([-0.2 log(1500)])
xticks( log(N0(:,1)));
xticklabels(N0(:,1));
xlabel('inoculum (cell/drop)');
ylabel('mean lag time (h)');
title(['N0=1 noise SD=' num2str(SDNoise) ])
box('on')
grid('on')
set(gca,'LineWidth',4)
set(gca,'GridAlpha', 0.5)
set(gca,'FontSize',60)
hold off
```

```
%%%The following code corresponds to the simulation producing the Fig. 4 related to growth activator%%%

close all
%clear all
```

```
Vdrop = 4.4e-4; %ml

Ndrop=1000;
inocula = logspace(0,3,10); %range of inocula to simulate
dt = 1/60; %heures
timeSpan = 0:dt:30; %heures

Nthresh = 1.6e8; %cell/ml. Threshold to calculate the lag time like in the experiments.

stdNoise = 0.88; %SD of the noise 0.87 with lambda and 2 sigma for the calib
stdNoiseTitle = stdNoise;
MM = 6.8246; %mean value of the Exp distribution of lag for logn
VV = 1.3322^2 - stdNoise^2; %std of the corrected distribution of the experimental lag that gollows a logn
%calculate the new param of the logn to remove the noise
mu = log(MM^2 / sqrt(MM^2 + VV));
s = sqrt(log(VV/MM^2 + 1));

mg = 0.8430; %average growth rate %1/h from inoculum 1
vg = 0.02; %SD growth rate from inoculum 1

spanThActv = logspace(-5,2,11);

rActv = mg/log(2); %the rate of production of growth activator is the inverse of the doubling time of cells.

%% synchronistation demonstration of the synchronisation effect
clear lagPop stdLagPop  decsyncPop leadsyncPop

tmes = zeros(Ndrop,length(inocula));
k=0;
for thActv = spanThActv
    k = k +1;
    clear tmes tsync tstsdsync decsync dec lead leadsync

    dec = ones(Ndrop,length(inocula))*nan;
    lead = ones(Ndrop,length(inocula))*nan;
    tmes = ones(Ndrop,length(inocula))*nan;

    for inoc = 1:length(inocula)

        for i = 1: Ndrop

            r = round(poissrnd(inocula(inoc))); %draw a random inoculum size according to the poisson distribution.
            if r~=0
                timeSeries = ones(r,length(timeSpan))*nan; %time serie of the growth for each bacterial lineage of this droplet
                timeSeriesActv = zeros(r,length(timeSpan)); %time serie of the growth activator produced by each bacterial lineage in this droplet
                clear tlag
                %lognormal
                tlag = lognrnd(mu,s,r,1); %draw randomly the cell lag according to the corrected cell lag distribution measured Fig. 2A.
                grate = normrnd(mg,vg,r,1); %draw randomly the cell growth rate according to the growth rate distribution measured Fig. 10.


                timeSeriesActv = exp(grate.*(timeSpan-tlag))-1; %production of molecule is linear with number of cells so it follows the exp growth
                timeSeriesActv(timeSeriesActv<0) = 0; % molecule concentration cannot be negative.
                timeSeriesActv = (timeSpan-tlag).*rActv.*timeSeriesActv; %multiplication by time and production rate.

                %calculate the total concentration of molecule produced by all
                %the cell in the droplet. Need of condition for drop with one cell
                %(no need to sum over cells)
                if r>1
                    actv = sum(timeSeriesActv);
                else
                    actv = timeSeriesActv;
                end
```

```
                %find the time at which the concentration of molecule gets
                %above a given threshold.
                tActv = timeSpan(find(actv>thActv,1,'first'));

                dec(i,inoc) = tActv - min(tlag); % difference of lag time of the leader cell and the lag time due to production of molecule.
                lead(i,inoc) = sum(tlag<=tActv);


                tlagActv = tlag;
                tlagActv(tlagActv>tActv)=tActv; %every cells lag time end when the molecule gets above the threshold.


                timeSeries(:,:)= exp(grate.*(timeSpan-tlagActv)); % proceed to the exponential growth of every bacterial lineage of this droplet.
                timeSeries(timeSeries<1)=1; % the lineage time series must start at 1 before the division of the bacteria


                %calculation of the cell concentration in this droplet along the
                %time by summing the time serie of bacterial lineage
                l = size(timeSeries);
                if l(1)==1
                    totDrop = timeSeries/Vdrop;
                else
                    totDrop = nansum(timeSeries)/Vdrop;
                end

                %measure the lag of the droplet by finding the time at which the
                %cell concentration gets above Nth like in the experiments
                tau = timeSpan(find(totDrop>Nthresh,1,'first'));
                if isempty(tau)
                    tmes(i,inoc) = nan;
                else
                    tmes(i,inoc) = timeSpan(find(totDrop>Nthresh,1,'first'))-log(Nthresh*Vdrop/r)/nanmean(grate);
                end
            else
                tmes(i,inoc) = nan;
            end
        end
    end

    for i = 1:length(inocula)
        tsync(i) = nanmean(tmes(:,i));
        tstdsync(i) = nanstd(tmes(:,i));
        decsync(i) = nanmean(dec(:,i));
        leadsync(i) = nanmean(lead(:,i));
    end

    lagPop(k,:)=tsync;
    stdLagPop(k,:)=tstdsync;
    decsyncPop(k,:)=decsync;
    leadsyncPop(k,:)=leadsync;
end

%%
close all


Y = repmat(transpose(log10(spanThActv)),1,10);
X = repmat(log10(inocula),11,1);
Z = lagPop;

%population lag time vs threshold and inoculum
figure('Renderer', 'painters', 'Position', [10 10 900 900]),
surf(X,Y,Z);
```

```
%title('population lag time')
xlabel('inoculum')
xticks(log10(round(inocula)))
xticklabels(round(inocula))

ylabel('threshold')
yticks(log10(spanThActv(1:2:end)))
yticklabels(num2str(transpose(spanThActv(1:2:end)),'%1.0e'))
zlabel('population lag time')
c = colorbar;
set(gca,'FontSize',30)
c.Location='northoutside';
view(35.207879105520632,39.388548057259705)

% population lag time minus leader cell lag time
figure('Renderer', 'painters', 'Position', [10 10 900 900]),
Y = repmat(transpose(log10(spanThActv)),1,10);
X = repmat(log10(inocula),11,1);
decsyncPop(decsyncPop<=dt)=0;
Z = decsyncPop;

surf(X,Y,Z);

%title('time difference between cell leader lag time and lag time of population')
xlabel('inoculum')
xticks(log10(round(inocula)))
xticklabels(round(inocula))

ylabel('threshold')
yticks(log10(spanThActv(1:2:end)))
yticklabels(num2str(transpose(spanThActv(1:2:end)),'%1.0e'))
zlabel('lag pop - lag leader cell')
c = colorbar;
set(gca,'FontSize',30)
caxis([0, 2]);
c.Location='northoutside';
view(35.207879105520632,39.388548057259705)

%number of cells that multiply before synchro
figure('Renderer', 'painters', 'Position', [10 10 900 900]),
Y = repmat(transpose(log10(spanThActv)),1,10);
X = repmat(log10(inocula),11,1);
Z = leadsyncPop;

surf(X,Y,Z);

%title('number of cell leaders')
xlabel('inoculum')
xticks(log10(round(inocula)))
xticklabels(round(inocula))

ylabel('threshold')
yticks(log10(spanThActv(1:2:end)))
yticklabels(num2str(transpose(spanThActv(1:2:end),'%1.0e'))
zlabel('number of leader cells')
set(gca,'FontSize',30)
c = colorbar;
caxis([1 5]);
c.Limits = [1 5];
c.Ticks = [1 2 3 4 5 6 7 8];
c.Location='northoutside';
view(35.207879105520632,39.388548057259705)
```

To add a 0.43 correlation between growth rate and lag time the code above can by modify with this snippet in two location. The homemade function that is called is given below.

```
tlag = lognrnd(mu,s,r,1); %draw randomly the cell lag according to the corrected cell lag distribution measured Fig 2A.
grate = normrnd(mg,vg,r,1); %draw randomly the cell growth rate according to the growth rate distribution measured Fig. 10.

correl = 0.43
X= rand_LogN_Norm_Correl(MM, VV, mg, vg, correl ,r); %draw randomly the lag time and growth rate correlated as in the experiment.
tlag = X(:,1);
grate = X(:,2);
```

```
function X = rand_LogN_Norm_Correl(meanLogN, stdLogN, meanNorm, stdNorm, correl,n)

muconv = @(m,v) log(m/sqrt(1+v/m^2)); %convert mean and std of logN in its 1st parameter
sigmaconv = @(m,v) sqrt(log(1+v/m^2)); %convert mean and std of logN in its 2sd parameter

Z = mvnrnd([0 0], [1 correl; correl 1], n);
U = normcdf(Z,0,1);
X = [logninv(U(:,1),muconv(meanLogN,stdLogN),sigmaconv(meanLogN,stdLogN)) norminv(U(:,2),meanNorm,stdNorm)];
end
```
